# Pedigree-based random effect tests to screen gene pathways

**DOI:** 10.1186/1753-6561-8-S1-S100

**Published:** 2014-06-17

**Authors:** Marcio Almeida, Juan M Peralta, Vidya Farook, Sobha Puppala, John W Kent, Ravindranath Duggirala, John Blangero

**Affiliations:** 1 Department of Genetics, Texas Biomedical Research Institute. 7620 NW Loop 410, San Antonio, TX 78245, USA; 2 Centre for Genetic Epidemiology and Biostatistics, University of Western Australia, WA, Australia

## Abstract

The new generation of sequencing platforms opens new horizons in the genetics field. It is possible to exhaustively assay all genetic variants in an individual and search for phenotypic associations. The whole genome sequencing approach, when applied to a large human sample like the San Antonio Family Study, detects a very large number (>25 million) of single nucleotide variants along with other more complex variants. The analytical challenges imposed by this number of variants are formidable, suggesting that methods are needed to reduce the overall number of statistical tests. In this study, we develop a single degree-of-freedom test of variants in a gene pathway employing a random effect model that uses an empirical pathway-specific genetic relationship matrix as the focal covariance kernel. The empirical pathway-specific genetic relationship uses all variants (or a chosen subset) from gene members of a given biological pathway. Using SOLAR's pedigree-based variance components modeling, which also allows for arbitrary fixed effects, such as principal components, to deal with latent population structure, we employ a likelihood ratio test of the pathway-specific genetic relationship matrix model. We examine all gene pathways in KEGG database gene pathways using our method in the first replicate of the Genetic Analysis Workshop 18 simulation of systolic blood pressure. Our random effect approach was able to detect true association signals in causal gene pathways. Those pathways could be easily be further dissected by the independent analysis of all markers.

## Background

The whole genome sequencing (WGS) platforms are changing the "game" in several scientific fields by offering a cost-effective way to harvest all genetic information of an individual or populations of interest. Genome-wide association studies (GWAS) approaches have been widely used in the identification of genetic markers associated with phenotypes of interest based on the common variant-common disease assumption [1]. The results of epidemiologic GWAS, while successful in the sheer number of quantitative trait loci that have been localized, have been relatively disappointing in their failure to identify the underlying causal genes for complex diseases like cancer, hypertension, and diabetes [1,2]. Generally, the significantly associated single-nucleotide polymorphisms (SNPs) are responsible for a very small proportion of these traits' heritability; several sources of this missing heritability have been proposed, with rare variation notable among them. WGS platforms, when applied to a large human pedigree, offer a unique possibility to study rare variant segregation and its role in a phenotype of interest [3]. Rare variants are clear candidates to explain part of the missing heritability paradox, but the low number of copies requires the development of alternative approaches to account for their association [2]. More importantly, rare variants of larger effect are far more likely to rapidly lead to causal gene identification than common variants of very small effect.

A *gene pathway *is an organized group of genes acting together to perform a specific cellular or physiological function. This high level of organization allows a fast and accurate response to any insult that a cell or tissue would suffer. This information is used for candidate genes annotation and several databases offer pathways information like GO (Gene Ontology) and KEGG (Kyoto Encyclopedia of Genes and Genomes) [4,5]. The causal component of a gene pathway will involve all phenotypically relevant functional variants of the genes in the pathway. To develop an omnibus test of whether a gene pathway plays a role in influencing a given phenotype, we must aggregate information into a single parameter.

A standard way to examine genetic variation across individuals is to examine genetic relationship matrices (GRMs) that quantify pairwise genetic similarity by the number of shared IBD (identical-by-descendent) or IBS (identical-by-state) alleles. Standard quantitative genetic methods use IBD-derived coefficients to structure pairwise phenotypic covariances to obtain estimates of overall heritability and linkage [6]. Here, we present a new approach that uses a pathway-specific GRM (PSGRM) for each available KEGG pathway to structure a pathway-specific variance component. A given PSGRM is estimated using the information provided by the complete set of sequence variant in or near genes of a specific gene pathway. Using the variance component model implemented in SOLAR, we employ a likelihood ratio test (LRT) of the PSGRM model against a null model only using the expected IBD matrix derived from the pedigree relationships [6]. PSGRM models that deviated from the null model and accounted for a significant portion of the trait's heritability are selected. Genes in those pathways that are shown to potentially harbor functional genetic variants could then be tested independently for causality. We also tested how minor allele frequency (MAF) spectra contribute to the PSGRM estimation. Our approach reduces the number of multiple independent hypotheses tested in a WGS and will reduce the computational burden. The explicit addition of a priori biological knowledge will aid candidate SNP annotation and interpretation of results from a WGS or GWAS study.

## Methods

### Genetic Analysis Workshop 18 data acquisition

Our analyses were performed on the Genetic Analysis Workshop 18 (GAW18) data set using simulated systolic blood pressure as our trait of interest [7]. All analyses were performed using the first simulated phenotype replicate (SIMPHEN.1.csv).

### KEGG database acquisition and gene mapping

The latest KEGG database release was obtained (10/2012). The database information was parsed and each gene assigned to its respective pathway. A given gene could be a member of more than 1 pathway. Each gene was mapped using the longest known splicing isoform using the latest RefSeq database (09/2012). For each mapped gene, we defined limits 5 kilobases (kb) upstream of the transcription start site and downstream of the stop codon, and all single-nucleotide variants located inside this region were selected. Using the sequencing data provided for GAW18, we collected all genotypes for those variants in all individuals. All variants within a gene were merged into a single pathway file.

### PSGRM estimation

All sequence variation in a gene pathway was used as an input to KING software [8] for the estimation of the PSGRM. KING uses an efficient algorithm implementation that allows fast kinship estimation for all pairs of individuals. The PSGRMs were calculated using the "robust" algorithm implementation, taking into account the potential for underlying genetic heterogeneity.

### Gene pathway burden test

A new variance component parameter was introduced into a standard pedigree-based variance component model $\text{Ω}=\sigma_{Total}^{\text{2}}\left(\text{2}\text{Φ}h_{r}^{\text{2}}+\text{2}Eh_{gp}^{\text{2}}+Ie^{\text{2}}\right)$, where *Ω *is the phenotypic covariance matrix, $\sigma_{Total}^{\text{2}}$ is the total phenotypic variance, $h_{r}^{\text{2}}$, $h_{gp}^{\text{2}}$, and $e^{\text{2}}$ respectively represent the proportion of $\sigma_{Total}^{\text{2}}$ that can be attributed to the residual additive effect of polygenes, the gene pathway-specific variation, and a random environmental effect. Several critical structuring kernels are employed to model the covariances between individuals: Φ is the expected or theoretical kinship matrix integrated over the genome, *E *is the empirically estimated pathway-specific kinship matrix (the PSGRM), and *I *is the identity matrix. Such kernel-based approaches for summarizing the effects of multiple genetic variants have been proposed decades previously [9] and has grown in popularity recently [10,11] Maximum likelihood estimates (assuming a multivariate normal probability density) and LRTs of the $h_{gp}^{\text{2}}$ parameter were obtained using an extension of the *polygenic *command in SOLAR independently for each gene pathway [11]. The significance of each gene pathways variance component estimate was obtained from a LRT against the null model $\text{Ω}=\sigma_{Total}^{\text{2}}\left(\text{2}\text{Φ}h_{r}^{\text{2}}+Ie^{\text{2}}\right)$. Because the variance component $h_{gp}^{\text{2}}$ is tested on its boundary, the LRT statistic is distributed as a 50:50 mixture of a 1-degree-of-freedom chi-square and a point of mass zero [12] although this is conservative [13]. For both models, we calculated the heritability of systolic blood pressure using the first simulated phenotypic replicate. We used sex, age, and smoking status as covariates in our model. To control for possible population stratification, we also included as covariates the first 5 genetic principal components calculated from a subset of ~29,000 low, mutual, linkage disequilibrium (LD) SNPs from the GAW18 GWAS data. Those principal components are responsible for 5% of the trait's total variance and were calculated in 117 founder samples using routine *prcomp *in R [14] and were then projected to the rest of the genotyped pedigree so as to not incorrectly capture systematic pedigree information.

## Results

### KEGG database organization and initial processing of GAW18 data

Initially, we downloaded the latest KEGG database release; this database is composed of 223 gene pathways for which we could classify 2428 genes located on the odd-numbered chromosomes. Each gene was mapped using the hg19 human genome assembly as a reference. Using the GAW18 WGS data set, we selected all variants near each gene (see Methods for details) of a gene pathway and all genotypes were collected. To further evaluate the sensitivity of the gene-pathway-specific LRT, we created 3 additional "virtual" pathways: (a) all causal genes used in the simulation, (b) major causal gene *MAP4 *and 9 randomly chosen genes, and (c) *MAP4 *plus 49 randomly chosen genes. The gene *MAP4 *exhibits the largest cumulative effect on the systolic blood pressure (SBP) phenotype and the number of random genes chosen mimics typical sizes of gene pathways.

### PSGRM estimation and testing

**Figure 1:**
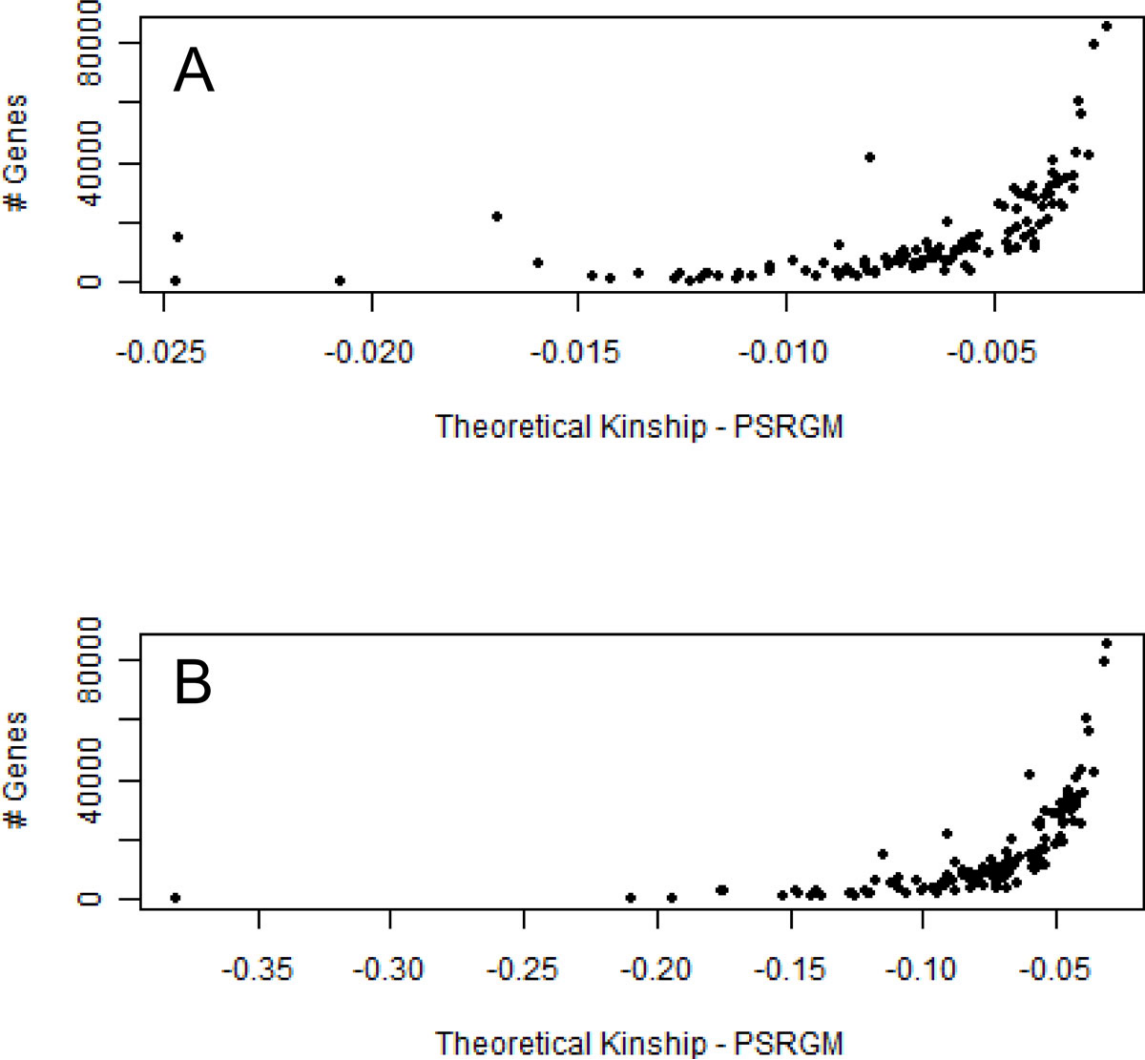
**Deviation of PSGRMs from the expected kinship matrix**. Dispersion plot comparing kinship estimators and the number of variants in a gene pathway. Panel A was constructed using the entire set of pairwise comparisons and panel B with only unrelated individuals.

All variants identified in a specific gene pathway were used as input for KING [8] to estimate a PSGRM using the robust routine to account for unknown population structure. This same procedure was applied in all gene pathways, including the virtual causal sets. As a proof of concept, the PSGRMs were compared to the pedigree-derived theoretical kinship by the comparison of the mean deviation of all pairwise kinship estimates. Figure 1A is a scatterplot showing the relationship between the number of genes in a pathway and the observed mean deviation for related individuals; Figure 1B shows the same comparison for unrelated individuals. Pathways composed by a larger number of genes tend to show smaller deviations as they asymptotically become more similar to the theoretical kinship estimator. Pathways composed by fewer genes have a more random behavior that illustrates the PSGRM capabilities of analysing relevant local patterns of allele sharing between individuals. As in traditional linkage analysis, deviation between the PSGRM and the theoretically expected genome-wide kinship matrix is required for the test to have power. When the 2 panels of Figure 1 are compared, the observed differences between the kinship estimates in unrelated individuals (1B) are more pronounced than the kinship estimates in Figure 1A. This result demonstrates that the PSGRM estimation captures potentially important information from the unrelated individuals as expected.

**Figure 2:**
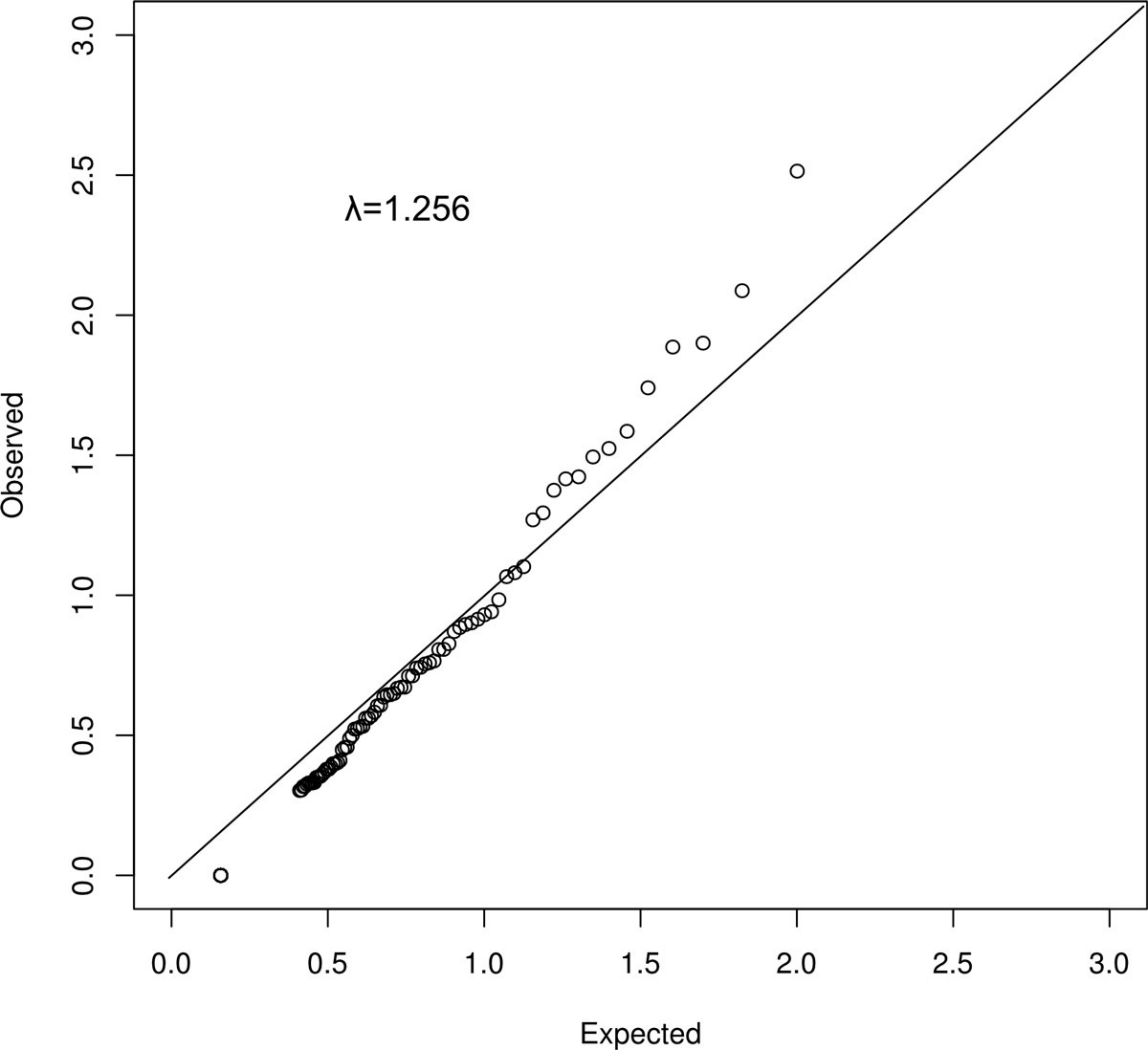
**Q-Q plot of PSGRM-based *p *values**. LRT results obtained from testing the addition of $h_{gp}^{\text{2}}$ term in the variance component model.

We estimated $h_{gp}^{\text{2}}$ for each PSGRM and tested whether the PSGRM can account for a significant proportion of trait's total heritability. The effect of each $h_{gp}^{\text{2}}$ term addition was tested using a LRT model implemented in SOLAR. All KEGG pathways were tested and *p *values obtained were used to create a Q-Q plot representation. Figure 2 depicts the Q-Q plot shows enrichment of signal with an observed inflation parameter of λ = 1.256. The GAW18 simulation offers a unique opportunity to test the sensitivity and specificity of our method. We created 3 "virtual" gene pathways composed totally or partially by causal genes that were used in the SBP phenotype simulation. The virtual gene pathways had their PSGRM estimated and their resulting $h_{gp}^{\text{2}}$ tested. The virtual pathways were able to absorb significant SBP heritability and the CAUSAL_SET made up of all causal genes shown to be the most significant pathway tested (Table 1). Two KEGG pathways, "cytokine_interaction" (2 causal genes in the GAW18 model) and "glutathione_metabolism" (2 causal genes), showed suggestive association. Two other associated pathways were "CAUSAL_1_TO_9" and "*E. coli *infection" that have 1 and 0 causal genes, respectively (ie, the latter is a false positive). Overall, the low false-positive ratio is encouraging, but the same test needs to be addressed across a larger number of traits. Our results show that the PSGRM ratio test was able to detect small genetic signals dispersed in larger gene pathway.

**Table 1:** Likelihood ratio tests of gene pathways.Results of likelihood ratio test using the pathway-specific variance component term $h_{gp}^{\text{2}}$. The table only shows pathways with *p*-values lower than 0.01.

Pathway	# Causal genes	Variance explained	$h_{r}^{\text{2}}$	$h_{r}^{\text{2}}$*p *value	$h_{gp}^{\text{2}}$	$h_{gp}^{\text{2}}$*p *value
CAUSAL_SET	15	0.2151	0.141	0.0019184	0.095	0.0000002
Cytokine_interaction	2	0.032	0.127	0.0130248	0.131	0.0006748
Glutathione_metabolism	2	0.027	0.221	0.0000136	0.074	0.00306
CAUSAL_1_to_9	1	0.0779	0.248	0.0000019	0.039	0.004348
*E. coli*_infection	0	0	0.244	0.0000099	0.051	0.0081785
CAUSAL_1_to_49	1	0.0779	0.265	0.0000131	0.038	0.178527

### Analysis of rare variant contribution to PSGRM testing

To assess the contribution of rare variants to phenotypes of interest, we selected and tested all variants in a pathway using 2 different MAF thresholds of 0.05 and 0.01 (Table 2). These low MAF spectrum variants typically did not improve causal pathway detection. Our focus on empirical IBD estimates contribute to this finding as there is less information regarding local IBD in the subset using only rarer variants. If we had employed a purely IBS-based kernel, these results might have been substantially different.

**Table 2:** Tests of $h_{gp}^{\text{2}}$ calculated using different frequency spectra. Association results of the CAUSAL sets using variants with different MAF spectra. The columns "Complete set", "MAF< 0.05" and "MAF 0.0" represent respectively the use all variants near genes, all variants with MAF< 0.05 and all variants with MAF < 0.01.

Gene set	Complete set	MAF <0.05	MAF <0.01
CAUSAL_1_to_49	0.178527	0.173653	0.0455696
CAUSAL_1_to_9	0.004348	0.000155	0.0111827
CAUSAL_SET	0.0000002	0.168555	0.0548205

## Discussion

WGS allows the complete enumeration of genetic variation. The sensitivity of WGS leads to the detection of a large number of rare variants not present in a standard high-density SNP array. Rare variants represent a partial explanation for the missing heritability paradox as their abundance and their expected genetic effect sizes are much higher than common variants [15]. Large extended pedigrees, like those in the GAW18 cohort, increase the probability of detecting rare variant effects, as variants that are rare in the population may be enriched by segregation in specific lineages [16]. Conventional genome-wide association-style multiple testing of the immense number of rare variants would require the use of stringent significance thresholds and reduce power. These barriers require the development of alternative methods to reduce the dimensionality of analysis. Here we present a hybrid method merging 2 approaches by calculating GRMs in predefined gene sets pathways. The definition of gene sets is flexible and allows the use of other sources of information, or even a candidate genes set defined by previous experiments. The PSGRM approach employed involves the genetic relationship estimation by analyzing local patterns of alleles shared in a gene pathway to reveal latent pathway-specific relationships across pedigrees and unrelated individuals. We focus on IBD estimation but many different IBS-related kernels could be utilized and should work better for studies that only have unrelated individuals focusing on association signal alone. Our IBD-based test is more similar to an extension of classical quantitative trait linkage but using empirical IBD estimates and generalizing them beyond a single genomic location. For the test to have power, there must be differences between the global IBD estimate and the pathway-specific estimate. The proposed single-degree-of-freedom test reduces the computational burden and allows a fast screen of gene pathways. The definition of a 5-kb region near a gene should be enable harvesting of a majority of regulatory elements playing a role in the expression regulation of the target gene; however, future studies should test larger distances also. For any empirically estimated GRM kernel, LD could be a source of bias and new approaches for calculating GRMs accounting for LD interference should be tested in the future [17].

## Conclusions

The analysis of WGS requires the development of new statistical and computational methods. Reduction in the number of hypotheses tested is critically important if reasonably sized samples of families are to identify causal genes. Our simple random-effect-based pathway-specific tests leads to potentially powerful tests of gene pathways that can then be further tested in more detail. The GAW18 simulated phenotypes offer a unique opportunity for methodological comparison and our method exhibited promising statistical features and can be easily extended to any other set of genes defined in an a priori manner.

## Competing interests

The authors declare that they have no competing interests.

## Authors' contributions

MA and JB designed the overall study; MA and JP conducted statistical analysis; MA, VF, SP, RD, JK, and JB drafted the manuscript. All authors read and approved the final manuscript.
